# Cavin gene family and caveolae-related disorders: pathogenetic roles and possible mechanisms

**DOI:** 10.1186/s12964-026-02716-3

**Published:** 2026-02-10

**Authors:** Youssef Osman, Nuraly S. Akimbekov, Emad M El-Zayat, Nourhan Hassan, Ilya Digel

**Affiliations:** 1 Institute for Bioengineering at FH, Aachen University of Applied Sciences, Jülich, Germany; 2 Department of Biotechnology, Al-Farabi Kazakh National University, Almaty, Kazakhstan; 3 Department of Biotechnology, Faculty of Science, Cairo University, Giza, 12613 Egypt

**Keywords:** Cavin, Cancer, Disorders, Mechanisms, Diagnostic markers, Therapeutic targets

## Abstract

Cavins, in concert with caveolins, orchestrate the formation and function of caveolae—specialized invaginations of the plasma membrane involved in mechanotransduction, lipid homeostasis, and cell signaling. The Cavin family comprises four members: Cavins 1–3, which are broadly expressed, and Cavin4, which is muscle-specific. Disruption of Cavin function via genetic mutations, epigenetic silencing, or altered expression is linked to a spectrum of caveolae-related disorders, including lipodystrophy, muscular dystrophies, insulin resistance, and cancer. This review offers a comprehensive analysis of the physiological roles, pathophysiological implications, and therapeutic potential of cavins, with emphasis on their involvement in cancer, metabolic diseases, and muscle disorders, highlighting their value as biomarkers and molecular targets in precision medicine. Specifically, Cavin1 serves as the central structural and functional scaffold of caveolae, linking mechanoprotection, lipid metabolism, and ribosomal RNA transcription to cellular stress adaptation and disease pathogenesis, whereas Cavin2 modulates caveolae morphology and signaling, with emerging roles in insulin sensitivity and inflammatory regulation. Cavin3, in turn, is considered a dynamic regulator of caveolae turnover and signal integration, linking caveolar function to cell signaling, DNA damage responses, and tumor suppression. Finally, Cavin4 plays a critical role in muscle-specific caveolae organization, mechanotransduction, and hypertrophic signaling. In the context of tumorigenesis, cavins together represent promising therapeutic targets due to their capacity to induce apoptosis, inhibit cancer cell migration and invasion, and modulate inflammatory responses; however, their roles appear to be context-dependent, with expression patterns and functional outcomes varying across tissue types.

## Introduction

The plasma membrane of many vertebrate cells, with the exception of neurons, can contain distinct, smooth invaginations known as *caveolae*. These structures are particularly abundant in adipocytes and endothelial cells, where they can occupy a significant portion (30–70%) of the membrane surface area [1–3]. Caveolae fulfill diverse roles in the cell: Their unique flask-like morphology and cytoskeletal associations provide mechanical stability to the cell membrane, modulate membrane fluidity and lipid composition, allowing cells to resist mechanical stress. Beyond these functions, caveolae act as specialized organizing platforms that compartmentalize, concentrate, and coordinate signaling molecules and receptors, thereby enabling precise regulation of intracellular signaling pathways [1, 4].

**Fig. 1:**
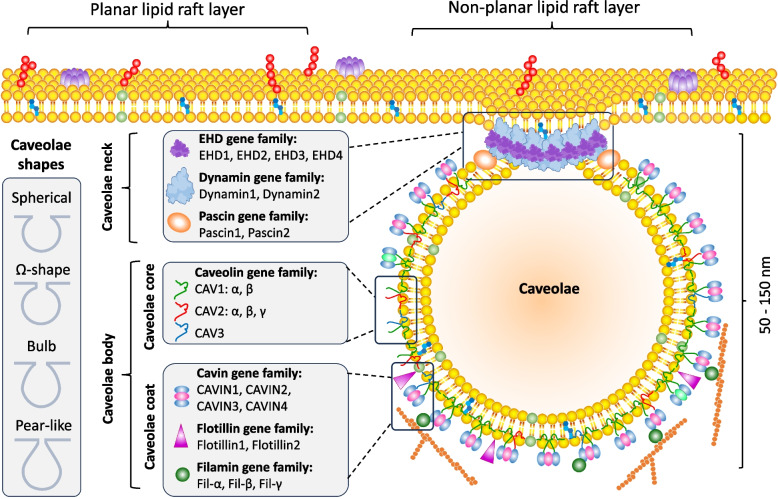
Shape diversity and the main structural components of caveolae. Left: schematic representation of characteristic shape diversity (spherical, Ω-shaped, bulb, and pear-like forms). Middle and right: principal molecular constituents of caveolae. Cavin family proteins (Cavin1–4), together determine caveolar curvature, stability, and dynamics. Additional caveolae-associated proteins, including EHD family members, dynamins, and pacsins, regulate membrane remodeling, scission, and caveolae trafficking at the neck region. Cytoskeletal linker proteins such as flotillins and filamins connect caveolae to actin and myosin filaments, contributing to mechanoprotection and signal integration

Unlike clathrin-coated vesicles, caveolae can be morphologically divided into two main regions (bulb and neck) and have a peculiar *cavin* coating. Cavins, together with caveolins, are crucial caveolae constituents. Caveolins scaffold the membrane to induce curvature, while cavins assemble into a peripheral coat that stabilizes the budded architecture and enables the mechanoprotective and signaling functions of caveolae [4, 5]. In addition, caveolae contain neck-associated proteins called EHD, the GTPase dynamin, and Pacsin2 (also known as Syndapin2), which play critical roles in regulating caveolae dynamics [6, 7]. Other important caveolae-associated proteins, Flotillin and Filamin, serve to link caveolae to the cytoskeleton, specifically to actin filaments and myosin. Transport proteins, such as Syntaxin and COPII, mediate the delivery of newly synthesized caveolins and cavins to the cell membrane (Fig. 1).

Growing evidence links caveolae protein dysfunctions to human diseases [4, 8–11] Understanding the molecular mechanisms that regulate the development, assembly, and disassembly of caveolar coats is essential for unraveling processes such as membrane remodeling, caveolar formation, cell signaling, and their involvement in diseases such as cancer, diabetes, lipid-related disorders, and muscular dystrophies.

Cavins, together with caveolins, are major caveolae constituents, determining their distinctive morphology and functional integrity [12, 13]. The Cavin protein family includes four members: Cavin1, Cavin2, and Cavin3 are broadly expressed across various cell types, while Cavin4 is restricted to muscle cells [14–17]. In addition to their obligatory presence in caveolae, Cavins can also be found in the cytoplasm and at the cell membrane [4]. While caveolins exist in multiple isoforms, Cavins (except for Cavin3) do not. However, relatively little is known about the mutations or methylation patterns of Cavin genes [14–17]. This knowledge gap underscores the need for further research into the genetic and epigenetic regulation of Cavins and their implications in cellular function and disease.

The aim of this study is to comprehensively review and discuss the roles of all four Cavin family members in disease, with a particular emphasis on cancer. By analyzing Cavin genetic loci, structural features, mutations, and functional mechanisms, we seek to elucidate their pathophysiological contributions and assess their potential as therapeutic targets.

### Structural properties and main functions of Cavins

Based on sequence and structural similarities, Cavins form a distinct protein family with four members:

- Cavin1 (chromosome 17q21.2),
- Cavin2 (chromosome 2q32.3),
- Cavin3 (chromosome 11p15.4), and
- Cavin4 (chromosome 9q31.1).

Each Cavin family member performs a distinct role depending on its structure, the morphology of the caveolae, and the specific cell type. Structurally, Cavins are organized into two helical regions (HR1 and HR2) and three disordered regions (DR1, DR2, and DR3) [12, 18, 19]. The helical regions, which are rich in negatively charged amino acids, show sequence homology and highly conserved α-helical secondary structures across all family members [9, 20]. According to Hansen and Nichols, Cavin orthologues are conserved only in vertebrates, likely due to the presence of morphologically distinct caveolae [21].

Cavins share many common features, such as leucine zipper-like regions that mediate protein–protein interactions [14–17]. Another hallmark of the Cavin family is the presence of PEST motifs in the disordered regions, which are rich in proline, glutamic acid, serine, and threonine [22]. These motifs mark the proteins for proteolytic degradation and are critical for Cavin turnover and regulation [9, 23]. Extensive post-translational modifications, particularly phosphorylation, occur mostly within the disordered regions [9, 21, 24–26].

Cavins can self-assemble into homo-oligomeric or hetero-oligomeric complexes that form the protein coat on the cytoplasmic face of caveolae. Ludwig et al. [27] demonstrated that these Cavin complexes are distributed across the caveolar bulb membrane and referred to them collectively as a “true caveolar coat.” While Cavin2 and Cavin3 do not form hetero-oligomers with each other, the HR1 domain at the N-terminus of Cavin1 facilitates both trimeric homo-oligomerization and the formation of hetero-complexes with either Cavin2 or Cavin3 [9, 24, 28, 29]. Cavin1 homo-trimers are of particular importance, as they are capable of promoting caveola formation independently of other Cavin proteins [9, 24, 28]. It was also proposed that HR2 can adopt a trimeric coiled-coil structure, possibly dependent on HR1 [24].

Despite ongoing research, many details regarding Cavin assembly into the spiral or striped patterns characteristic of caveolar coats remain unclear. It is known, however, that specific intermolecular interactions stabilize the monomeric Cavin components.

Collectively, Cavins are primarily responsible for governing the shape, function, and organization of caveolae and for modulating Caveolin behavior [30]. Although Cavins influence Caveolin availability, their main role is as scaffolding proteins that support caveolar structure and stability [8, 31].

## CAVIN1

### Cavin1: gene and protein

Cavin1 is the first and most prominent member of the Cavin family. Cavin1 is a specific and ubiquitously expressed caveolar protein, predominantly located on the cytosolic face of caveolae, but also found in the nucleus and associated with the plasma membrane [32, 33]. Due to its ubiquitous expression and essential role in caveola formation across various cell types, it is often referred to as the “gatekeeper Cavin” [9, 34]. Cavin1 is particularly abundant in adipose tissue, smooth muscle cells [35], and lung tissue, with lower expression levels detected in the endometrium, prostate, and placenta [21, 36, 37].

Originally, Cavin1 was known as Polymerase I and Transcript Release Factor (PTRF). Other synonymous names include FKSG13, Cavp60, and Branch Point Bridging Protein (BBP) [38, 39]. It has also been sometimes referred to as BFCOL1 (Binding Factor of a Type-1 Collagen Promoter [40]. These multiple names reflect its multifunctional roles in diverse cellular processes.

**Fig. 2:**
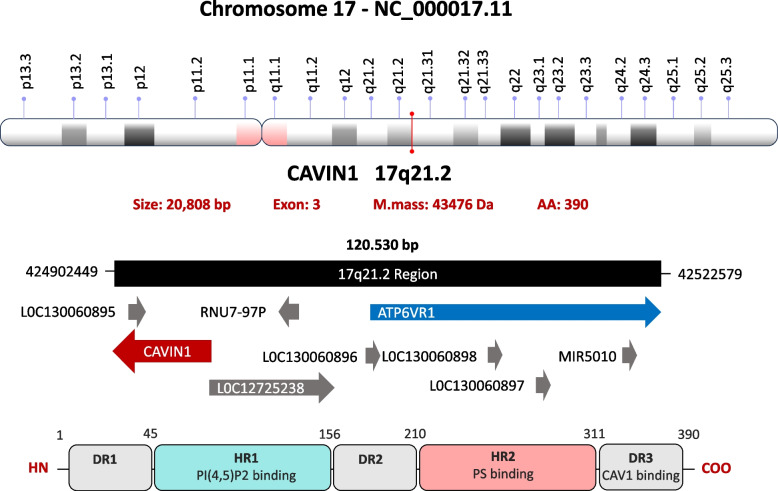
Structure of the Cavin1 gene and its transcription product. The upper panel shows the chromosomal localization of CAVIN1 gene (also known as PTRF) on chromosome 17 at band 17q21.2, including its genomic neighborhood and orientation within the locus. The gene consists of three exons, giving rise to a transcript encoding a protein of 390 amino acids with a molecular mass of ~ 43.5 kDa (NCBI Gene ID: 284,119). The lower panel illustrates the domain organization of the Cavin1 protein, displaying two conserved helical regions (HR1 and HR2) flanked by three intrinsically disordered regions (DR1–DR3). HR1 is involved in phosphatidylinositol-4,5-bisphosphate [PI(4,5)P₂] binding, whereas HR2 mediates phosphatidylserine (PS) binding. The C-terminal disordered region (DR3) contributes to interactions with caveolar components, including Caveolin1

The protein is encoded by the PTRF gene, which is located on the long arm of chromosome 17 (17q21.2) [16, 40]. The schematic structure of cavin gene is shown in Fig. 2.

The Cavin1 gene spans 20,808 base pairs and consists of three exons, which together encode a protein comprising 390 amino acids with a molecular mass of approximately 43.5 kDa [16]. Structurally, Cavin1 contains two helical regions – HR1 (111 amino acids) and HR2 (101 amino acids) – as well as three disordered regions: DR1 (45 amino acids), DR2 (54 amino acids), and DR3 (79 amino acids) [41].

Numerous studies have shown that the HR1 domain of Cavin1 facilitates both the trimeric homo-oligomerization of Cavin1 and the formation of heterocomplexes with Cavin2 or Cavin3 in solution [24, 28]. This domain is essential for the structural integrity and functional versatility of Cavin1, underscoring its pivotal role in caveolae dynamics and cellular signaling [23, 24].

### Functions of Cavin1

As a primary constituent of caveolae, Cavin1 is critical for their formation, maintenance, and dynamic regulation [42–44]. An increase in caveolar invagination is associated with elevated Cavin1 levels, whereas its dissociation leads to membrane flattening and reduced interaction with the cytoskeleton.

Wang et al. showed that Cavin1 acts as a signal transduction molecule involved in insulin signaling and the remodeling of membrane architecture, including the formation of focal adhesion complexes (FACs) and the dynamic regulation of caveolae [45]. In adipocytes, for example, insulin stimulation induces Cavin1 phosphorylation, promoting its detachment from caveolae and translocation to the nucleus. This process facilitates membrane stretching, which enhances lipid uptake by fat cells [45]. Despite these insights, the molecular mechanisms underlying Cavin1 disassembly at the membrane remain incompletely understood.

In vitro studies have shown that Cavin1 can induce membrane invaginations by binding to phosphatidylinositol 4,5-bisphosphate (PI(4,5)P₂) and phosphatidylserine (PS) [18, 43]. Positively charged residues such as Lys115 and Arg117 contribute to membrane curvature through electrostatic interactions between disordered regions (DRs) and helical regions (HRs) [23]. Phosphorylation is believed to weaken these interactions, promoting detachment from the membrane. In addition, phosphorylated regions of Cavin1 may serve as sites for phosphorylation-dependent proteolysis [46].

Cavin1's role in lipid metabolism appears to extend to the level of gene regulation; studies suggest it can enhance transcription rates in a concentration-dependent manner [47, 48]. Together with Caveolin1, Cavin1 acts as a mechanoreceptor and a key regulator of adipose tissue metabolism and expandability [49]. Its interactions with FACs influence interactions of plasma membrane proteins with the extracellular matrix. Caveolin1 and Cavin1 act synergistically also to create a unique lipid environment within caveolae [13]. These pathways may involve the activation of Golgi-associated kinase 1 A (GASK1A), linking caveolae biogenesis to lipid homeostasis. Cavin1 has also been associated with Mitsugumin 53 (MG53), also known as TRIM72, a member of the tripartite motif-containing (TRIM) protein family that acts as a docking protein during acute cell injury and membrane repair [35, 50–52].

### Cavin1-related disorders

The central role of Cavin1 in lipid regulation, both directly and through gene expression, will be further discussed in the context of clinical manifestations related to Cavin1 dysfunction, including genetic and sporadic disorders. Table 1 shows the prevalence of the most significant Cavin1-related disorders and the corresponding patterns in the Cavin1-dynamics.

**Table 1:** Major sporadic/metabolic disorders associated with Cavin1 and their prevalence

Disease	Worldwide prevalence (recent estimate)	Reference for prevalence	Cavin1 association
Diabetes mellitus	~ 828 million adults (global estimate for 2022)	Lancet/NCD-RisC global analysis [53]	Deficit/loss of function
Asthma	~ 262 million people (global estimate for 2019)	WHO asthma fact sheet [54]	Context-dependent dysregulation
Heart muscle disease	Rare–uncommon overall ~ 0.03–0.2%	Herr et al. [55]	Deficit/loss of function
Skeletal muscle dystrophy	Depends on the DMD type. For Duchenne type ~ 2 per 100,000 males	Kariyawasam et al. [56]	If applicable: deficit/loss of function
Lipodystrophy (CGL4 in particular)	Very rare; congenital generalized lipodystrophy overall ~ 1–10 per 1,000,000	Akinci et al. [57]	Deficit/loss of function

Although many of the diseases discussed in this review are highly prevalent and of major clinical importance, the roles of cavins have often been more thoroughly investigated and mechanistically characterized in less prevalent disorders. Within each cavin-specific chapter, sections are organized to progress from more prevalent diseases to those of lower prevalence or more limited clinical relevance.

### Cavin1 and sporadic/metabolic disorders

#### Cavin1 and diabetes mellitus

Insulin resistance, one of the primary causes of type 2 diabetes, results from defective insulin signaling in target tissues. Although there are animal and human studies showing that the absence of caveolae is associated with insulin resistance, direct evidence linking caveolae dysfunction to the pathophysiology of insulin resistance in type 2 diabetes and obesity remains limited [58].

Cavin1 is highly expressed in human adipocytes, where it localizes to the plasma membrane and caveolae [59]. Its subcellular localization is regulated by insulin, which induces its translocation from caveolae to the nucleus and cytoplasm [60, 61]. The insulin-induced redistribution of Cavin1 strongly supports its involvement in dynamic signal regulation and membrane remodeling. For example, Cavin1 has been shown to bind selectively to a subclass of caveolae involved in triacylglycerol metabolism, where it co-localizes with hormone-sensitive lipase (HSL). In response to insulin, both HSL and Cavin1 translocate into the cytosol. In many cells, Cavin1 is involved in transcription termination, interacting with thyroid transcription factor 1 and RNA polymerase I, and specifically binding the 3′-end of pre-rRNA transcripts [60]. Cavin1 may also act as a signaling mediator through its interaction with Paxillin [45]. This dual role of Cavin1 – as both a structural component and a signal transduction molecule – underscores its importance in insulin signaling and membrane remodeling. These functions include the formation of FACs and the dynamic reorganization of caveolae, which are critical for adipocyte membrane expansion and lipid accumulation – key elements for maintaining insulin sensitivity.

**Fig. 3:**
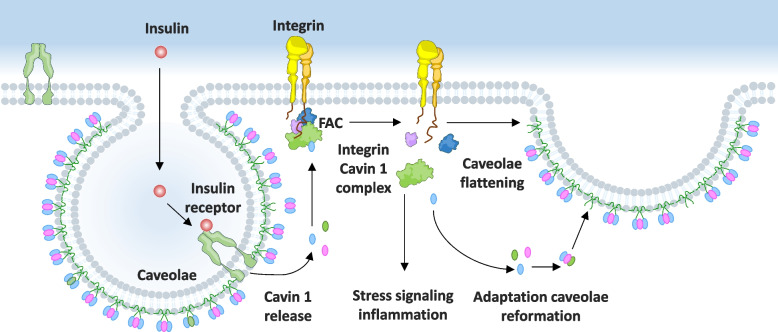
A model illustrating the proposed role of Cavin1 in insulin signaling, mechanotransduction, and stress responses relevant to diabetes mellitus. Under basal conditions, insulin binding to the insulin receptor promotes the translocation of Cavin1 from caveolae to focal adhesion complexes (FACs), where it supports their assembly and remodeling. Impairment of this trafficking destabilizes FACs and triggers downstream stress-associated signaling pathways, potentially contributing to inflammation and fibrotic remodeling observed in hypertrophic and obese adipocytes

According to the model proposed by Wang et al., under physiological conditions, insulin promotes Cavin1 translocation from caveolae to focal adhesions, facilitating their formation and remodeling. Loss of this remodeling response due to caveolae deficiency imposes mechanical stress directly on FACs and activates stress response [52]. This mechanism is schematically shown in Fig. 3. The model highlights Cavin1 as an important molecular link between caveolae dynamics, insulin responsiveness, and disease pathogenesis.

Downstream pro-apoptosis and pro-inflammatory pathways in adipose tissue (via focal adhesion kinase signaling) cause adipocyte dysfunction and ultimately result in lipodystrophic phenotype observed in patients with loss-of-function mutations in Caveolin1 or Cavin1, as well as in animal models lacking caveolae [62]. Many factors, including insulin stimulation, mechanical stress, or inflammatory cues can trigger partial caveolar flattening and the release of Cavin1 from the caveolar coat into the cytoplasm. During prolonged or excessive stress, caveolae flattening contributes to membrane adaptation, followed by reassembly once homeostasis is restored.

The discovered mechanisms related to insulin-induced Cavin1 translocation highlight the need for further research into its role in diabetes mellitus, including type 1 diabetes in infants. Understanding the other aspects by which Cavin1 regulates insulin signaling and adipocyte homeostasis could provide valuable insights into the pathogenesis of metabolic disorders and aid the development of novel therapeutic strategies.

#### Cavin1 and asthma

One of the disorders linked to Cavin1 dysfunction or loss is bronchial asthma, a chronic inflammatory disease characterized by airway inflammation, exacerbate type 2 immune responses and airway hyperresponsiveness [63]. Loss of Cavin1 also induces morphological changes in lung tissue in mice, including interstitial thickening, hypercellularity, increased collagen deposition, and infiltration by macrophages and CD45⁺ immune cells [64].

Cavin1 plays a regulatory role in the release of interleukin-33 (IL-33), a key cytokine in asthma pathophysiology, often referred to as an epithelial alarmin. IL-33 is usually stored in the nucleus and passively released during necrosis or asthma exacerbations. While the mechanisms by which viable cells release IL-33 are not fully understood, Cavin1 does not mediate this release directly but is essential in controlling the process [65]. In bronchoalveolar lavage fluid, when Cavin1 was partially lost, increased IL-33 release, but not IL-25, has been observed, indicating a specific regulatory role for Cavin1 [63].

Molecular mechanism of Cavin1 action in asthma presumably involves tyrosine phosphorylation or dephosphorylation in response to various extracellular signals. Notably, dephosphorylation of Cavin1 appears to reduce asthma exacerbations by limiting IL-33 release. For instance, in human bronchial epithelial cells (16HBE), Cavin1 is predominantly dephosphorylated at the Tyr158 site upon stimulation by lipopolysaccharide (LPS) or house dust mite (HDM) exposure – both known triggers of immune responses – facilitating Cavin1 translocation to the nucleus [63]. LPS, a microbial pattern recognition ligand, activates innate immune cells to eliminate pathogens. HDM, one of the most common triggers of respiratory allergies, stimulates dendritic cells (DCs) and plays a central role in initiating adaptive allergic immune responses.

#### Cavin1 in skeletal and heart muscle dystrophy

Cavins and Caveolins participate in the regulation of myocardial function and the heart’s response to mechanical and metabolic stressors, such as ischemia [66]. Among these, Cavin1 is particularly important in muscle physiology, as mutations in the Cavin1 gene lead to a significant reduction in caveolae formation in both cardiac and skeletal muscles, contributing to the development of cardiac hypertrophy [67].

Patients with CGL4, who carry homozygous Cavin1 mutations, as described in the previous section, may also present with long-QT syndrome and fatal cardiac arrhythmias (e.g., Romano-Ward syndrome) [68]. The pathogenic effects of Cavin1 deficiency are attributed to a decrease in caveolae density and reduced expression of Caveolin-3 (Cav3), the muscle-specific caveolin isoform, in skeletal muscle tissue.

Studies using Cavin1 knockout (Cavin1⁻/⁻) mouse models have confirmed that Cavin1 deletion results in the loss of caveolae and reduced expression of both Caveolin-1 and Caveolin-3 in cardiac tissue. These animals exhibit impaired cardiac ejection, along with altered myocardial and coronary responses to mechanical stretch and ischemia–reperfusion injury. However, the precise effects of Cavin1 deficiency on cardiac morphology and electrical conduction remain incompletely understood [69].

Further research has shown that Cavin1 is necessary to maintain the structural integrity and mechanical responsiveness of cardiomyocytes [66]. In its absence, cardiomyocytes become more susceptible to damage under mechanical stretching and ischemic stress. Normally, caveolae function as mechanosensors that buffer mechanical stress by modulating membrane tension. In Cavin1-deficient hearts, this protective mechanism is compromised, increasing the risk of mechanical injury, cardiac hypertrophy, and arrhythmias [70].

Cavin1 deficiency also negatively affects nitric oxide (NO) signaling in the heart. Caveolae compartmentalize endothelial nitric oxide synthase (eNOS), which synthesizes NO, a critical regulator of vascular tone and cardiac function [71]. In Cavin1-deficient hearts, the loss of caveolae disrupts eNOS localization and activity, leading to reduced NO production and endothelial dysfunction [30]. This further exacerbates cardiac impairment and promotes pathological remodeling of the heart.

#### Cavin1 and lipodystrophy

Congenital generalized lipodystrophy (CGL) is a rare autosomal recessive disorder, affecting approximately one in ten million people worldwide [72]. Also known as Berardinelli–Seip syndrome (BSCL), Brunzell syndrome, Seip syndrome, or total lipodystrophy, CGL is characterized by a near-complete absence of adipose tissue from birth, leading to the development of metabolic complications such as hypertriglyceridemia, diabetes mellitus, and hepatic steatosis later in life.

CGL is classified into four subtypes (CGL1–4), based on mutations in the following genes [73, 74]:

- AGPAT2 (CGL1, 9q34.3),
- BSCL2 (CGL2, 11q13),
- Caveolin1 (CGL3, 7q31.1), and
- Cavin1 (CGL4, 17q21.2).

In some CGL cases, no mutations in these genes have been identified, suggesting the involvement of sporadic events or yet-unknown genetic factors that warrant further investigation [75, 76].

Recent studies have provided deeper insights into CGL type 4 (CGL4). Valentina Mancioppi and colleagues [77] described pediatric twins with CGL4 carrying a novel homozygous mutation in exon 1 of the Cavin1 gene. In another report, Ardissone et al. identified a different Cavin1 mutation in a child with very mild congenital lipodystrophy combined with mild myopathy [78]. Unlike other CGL types, CGL4 (caused by loss-of-function mutations in Cavin1) presents a distinct phenotype involving generalized lipodystrophy, muscular dystrophy, and cardiac anomalies [79, 80]. Additional features include severe lipid dysregulation and abnormal lipid storage, as documented in multiple studies [73, 80–84].

Although Cavin1 is known to positively regulate lipolysis in adipocytes, the molecular mechanisms underlying this role remain poorly understood [49]. Disruptions in lipolysis, as reported by Capozza et al. [85], lead to insulin resistance and ultimately to severe lipodystrophy, as demonstrated by Garg et al. [86] and Kim et al. [87].

In Cavin1 knockout mouse models, a significant reduction in adipose tissue is observed throughout the body, along with resistance to diet-induced obesity. Ding et al. [87] reported that the metabolic dysfunctions in adipose tissue, muscle, and liver in Cavin1-null mice closely resemble those seen in humans lacking caveolae, resulting in a pleiotropic phenotype. Reduced lipid accumulation in adipocytes appears to stem from impaired fatty acid uptake and incorporation, as well as near-complete inhibition of insulin-stimulated glucose transport [88, 89].

One newly described CGL4 patient exhibited diabetes mellitus, vitamin D deficiency, hypocalcemia, bilateral cataracts, and hyperuricemia. In adipocytes, lipolysis is initiated by hormone-sensitive lipase (HSL), which is activated through the phosphorylation of perilipins – members of a protein family that coats lipid droplets and protects them from premature breakdown [49, 90]. Catecholamines stimulate lipolysis by promoting phosphorylation of HSL and perilipins. This process is mediated by protein kinase A, which phosphorylates HSL at Ser563, Ser659, and Ser660, both in vitro and in primary rat adipocytes. Conversely, insulin suppresses lipolysis via activation of phosphodiesterase 3B, which reduces intracellular cAMP levels [91].

In addition to lipodystrophy, mutations in CGL4 are associated with progressive encephalopathy (with or without lipodystrophy, in autosomal dominant forms) and spastic paraplegia 17, highlighting the broader involvement of lipodystrophy genes in adipogenesis, glucose metabolism, and energy homeostasis [90, 91].

### Cavin1 and tumorigenesis

The role of Cavin1 in cancer is complex and context-dependent, with evidence supporting both tumor-suppressive and oncogenic functions, depending on the cancer type and molecular environment.

Many studies have shown that Cavin1 expression is downregulated in subsets of breast and lung cancers, and its loss is associated with tumor progression and poor prognosis [92–94]. In prostate cancer, Cavin1 has been shown to inhibit tumor growth by modulating signaling pathways that regulate cell proliferation and survival [95]. In contrast, in some other malignancies Cavin1 apparently exhibits oncogenic properties. For instance, Martinez-Val et al. evaluated Cavin1 expression levels within the tumor cells in colorectal carcinoma (CRC), and reported that Cavin1 is significantly overexpressed in patients who experienced disease relapse, suggesting that Cavin1 may contribute to tumor aggressiveness and treatment resistance [96]. Similarly, Yi et al. in an earlier study noticed that Cavin1 levels in cancer cells are often associated with multidrug resistance [97].

This apparent contradiction may stem from Cavin1’s involvement in diverse cellular processes, including membrane dynamics and integrity, signal transduction, and mechanoprotection, with all of them being crucial for cancer cell migration, invasion, and metastasis [98]. A complete picture of these interactions is still lacking; however, Cavin1 appears to engage in complex, non-linear regulatory relationships even with well-studied key proteins such as p53 [99] and Mdm2 [100] which are central to cell-cycle control and apoptosis.

Recent extensive studies by Low et al. and by Martinez-Val et al. revealed another interesting aspect of Cavin1 involvement in shaping the tumor microenvironment. In particular, Cavin1 expression has been associated with the regulation of tumor angiogenesis and immune cell infiltration, adding further complexity to its function in cancer biology [95, 96]. Additionally, cancer-specific epigenetic modifications, including DNA methylation and histone acetylation, may influence Cavin1 expression and contribute to its dual role in tumorigenesis in a complex manner [101, 102]. The prevalence of the most significant Cavin1-related cancers and corresponding changes in the Cavin1-dynamics are presented in Table 2.

**Table 2:** Major Cavin1-related cancers their prevalence

**Disease**	**Worldwide incidence (**age-standardised rates)	**Reference for prevalence**	**Cavin1 association**
Prostate cancer	29.4 per 100,000	World Cancer Research Fund [103]	Context-dependent/dual
Lung cancer (incl. LUAD)	23.6 per 100,000	World Cancer Research Fund [103]	Context-dependent (often ↓ in tumors
Colorectal cancer (CRC)	18.4 per 100,000	World Cancer Research Fund [103]	Mostly ↓
Liver cancer (incl. hepatocellular carcinoma)	8.6 per 100,000	World Cancer Research Fund [103]	Context-dependent/dual
Brain/CNS tumors (incl. glioblastoma)	3.5 per 100,000	World Cancer Research Fund [103]	Often ↑
Skin cancer (incl. cSCC)	3.2 per 100,000	World Cancer Research Fund [103]	Mostly ↓
Rhabdomyosarcoma (RMS)	0.5 per 100,000	Skapek et al. [104]	Context-dependent/dual

#### Cavin1 and prostate cancer

Prostate cancer is the fourth leading cause of cancer-related death globally, with approximately 1.41 million new cases reported annually [105, 106]. Prostate cancer primarily arises from glandular cells of the prostate acini, but also involves stromal cells and the extracellular matrix, which support tumor growth and metastasis. Tumor-derived extracellular vesicles contribute to tumor progression, although the molecular mechanisms that regulate their secretion and trafficking remain poorly understood.

Within this complex tumor microenvironment, Cavin1 has emerged as a key regulator of lipid metabolism and RNA polymerase I transcription in stromal cells, influencing ribosomal activity and transcriptional control [77]. Stromal Cavin1 modulates the tumor microenvironment by regulating lipid distribution and inflammatory signaling, thereby influencing metastasis [107–110]. Additionally, Cavin1 may function as an adipokine, potentially contributing to the deleterious effects of visceral fat accumulation – a known risk factor for aggressive prostate cancer [111]. Under physiological stress, such as fasting or exercise, triacylglycerol reserves stored in lipid droplets are mobilized as an energy source [112]. Because certain lipid species are cytotoxic, caveolar lipid absorption ma have a cytoprotective role. Due to these mechanisms, Cavin1 deficiency can substantially impact cellular energy homeostasis and alter the tumor microenvironment [113].

Interestingly, in prostate cancer, Cavin1 exhibits a dual expression pattern: it is upregulated in tumor epithelial cells but downregulated in tumor-associated stromal cells. For instance a recent study by Jin-Yih Low et al. showed the context-dependent role of Cavin1 in prostate cancer, demonstrating that reduced Cavin1 expression in epithelial tumor cells – but not stromal cells – is associated with disease progression, while restoration of Cavin1 expression mitigated tumor development [95].

Several functional studies showed that ectopic Cavin1 expression in prostate cancer cells reduces aggressive tumor traits, including proliferation, anchorage-independent growth, migration, invasion, lymphangiogenesis, and angiogenesis, both in vitro and in vivo [93, 114, 115]. Conversely, Cavin1 depletion in prostate stromal cells results in decreased lipid droplet formation and increased pro-inflammatory signaling. Collectively, these findings support the view that Cavin1 possesses tumor-suppressive properties in prostate cancer. High Cavin1 expression appears to impede cancer progression, whereas Cavin1 downregulation in tumor epithelial cells has been associated with increased malignancy and poor prognosis [95, 116].

#### Cavin1 and lung adenocarcinoma (LUAD)

Studies by Gamez-Pozo et al., later by Peng et al. and also recently by Mou et al. have shown that Cavin1 mRNA and protein expression are in significantly downregulated in lung tumor tissues compared to adjacent non-tumor tissues [117–119]. However, this effect probably should not be generalized to all lung cancers, because upregulation of Cavin1 mRNA was more frequently observed in lung adenocarcinoma (LUAD) (29.7%) than in lung squamous cell carcinoma (LUSC) (15.8%) [120].

Importantly, higher Cavin1 expression in LUAD correlates with advanced N-stage (lymph node involvement) and a higher pathological TNM stage, suggesting a strong link between Cavin1 and disease progression [120]. One proposed mechanism proposed by Hill et al.is that Cavin1 acts as a scaffold protein for Caveolin1, stabilizing its function by inhibiting internalization and preventing lysosomal degradation [43].

Cavin1 has also been implicated in regulating AKT phosphorylation, thereby influencing cell proliferation and survival [121]. Additional research has demonstrated that receptor tyrosine kinase-like orphan receptor 1 (ROR1) promotes the interaction between Cavin1 and Caveolin1, sustaining pro-survival signaling pathways in LUAD [122]. Disrupting this interaction may therefore represent a novel therapeutic strategy, with the potential to inhibit tumor progression and improve clinical outcomes in LUAD patients.

#### Cavin1 and colorectal tumors (CRC)

Colorectal cancer is the second leading cause of cancer-related deaths and the third most commonly diagnosed cancer worldwide [123]. It originates from the uncontrolled proliferation of abnormal epithelial cells in the colon or rectum, often leading to metastasis. The severity of CRC is clinically assessed using the TNM staging system (based on the Tumor size/invasion, Node involvement, and distant Metastasis). The disease is also classified into four consensus molecular subtypes (CMS1–CMS4), each defined by distinct molecular mechanisms with implications for prognosis and treatment response [124–126].

The role of Cavin1 in CRC remains context-dependent and is under active investigation. While some studies suggest a tumor-suppressive function, others implicate Cavin1 in tumor progression and therapy resistance. This suggests that the role of Cavin1 may vary depending on cell type, tumor microenvironment, or genetic background. For example, Ana Martinez-Val et al. used a three-dimensional (3D) in-vitro model of colorectal cancer to demonstrate that Cavin1 contributes to tumor cell invasiveness, epithelial-mesenchymal transition (EMT), and cell–cell adhesion [96]. These findings suggest that Cavin1 may accelerate tumor progression and contribute to treatment resistance not only in CRC, but also in glioblastomas and pancreatic cancers [97, 127–129].

However, other studies have shown that Cavin1 expression is significantly reduced in CRC tumor tissues. In the work by Wang et al. [130], ectopic expression of Cavin1 in colorectal cancer cell lines (Colo320, HT29, and CaCo2) inhibited their malignant potential. Notably, Colo320 cells, which express the lowest endogenous Cavin1 levels, showed the greatest reduction in proliferation and anchorage-independent colony formation upon Cavin1 overexpression. In xenograft models, Cavin1 overexpression led to significant tumor growth suppression in Colo320-derived tumors, while Cavin1 deletion in CaCo2 cells enhanced tumor growth. These results support a tumor-suppressive role for Cavin1 in CRC, potentially mediated by inhibition of the AKT/mTOR signaling pathway, a key regulator of cell growth, metabolism, and proliferation [127, 131].

Further complexity arises from retrospective clinical data showing that Cavin1 expression negatively correlates with both TNM stage and lymph node metastasis in CRC patients, indicating its potential as a prognostic biomarker [131].

Mechanistically, Cavin1 interacts with the PI3K/AKT/mTOR pathway, a central signaling axis in cancer biology. Wang et al. [130] demonstrated that Cavin1 overexpression suppresses this pathway, leading to reduced proliferation, migration, and invasion of CRC cells. These findings reinforce the hypothesis that Cavin1 may act as a tumor suppressor by regulating PI3K/AKT/mTOR signaling. However, the complex regulation of this pathway by various genetic and environmental cues underscores the need for further studies to define the precise molecular mechanisms involved. Clarifying these mechanisms will be crucial for assessing Cavin1’s potential as a therapeutic target or prognostic marker in colorectal and other cancers.

#### Cavin1 and hepatocellular carcinoma (HCC)

Hepatocellular carcinoma (HCC) is the sixth most common cancer globally and the third leading cause of cancer-related mortality, accounting for approximately 83,000 deaths annually [132]. Most HCC cases are diagnosed at intermediate or late stages, complicating early detection and effective treatment.

In a very recent study, Xingyuan Hao et al. have analyzed gene sets from The Cancer Genome Atlas (TCGA) using gene set enrichment analysis to explore the role of Cavin1 in HCC [132]. Their results revealed that Cavin1 is closely associated with the Wnt/β-catenin signaling pathway, a well-known driver of HCC progression [133]. In particular, overexpression of Cavin1 significantly suppressed the expression of key Wnt pathway molecules, including β-catenin, c-Myc, and matrix metalloproteinase 9. Conversely, Cavin1 deficiency led to upregulation of these proteins [132, 133].

Statistical analyses made by the same research team showed (a) that Cavin1 expression is lower in HCC tumors compared to adjacent non-tumor tissue and (b) that low Cavin1 levels correlated with poorer prognosis in HCC patients [132]. Functionally, Cavin1 overexpression inhibited cell migration, proliferation, and epithelial-mesenchymal transition (EMT) in HCC cells. In contrast, Cavin1-deficient HCC cells exhibited enhanced migration and invasiveness. In supplementary in vivo experiments using subcutaneous xenografts in nude mice, Cavin1 overexpression led to significantly smaller tumor size and weight, and fewer lung metastases compared to controls [132]. These effects further supported the tumor-suppressive role of Cavin1 in HCC.

However, regarding the Cavin1-role in HCC some conflicting findings exist – for example, Xu et al. reported opposing outcomes under different experimental conditions, indicating the need for further validation [133]. Deng et al. [134] reported that suppression of ths Wnt pathway can lead to paradoxical increases in c-Myc and β-catenin levels, emphasizing its complex regulation.

One of the hallmarks of EMT in HCC is the loss of E-cadherin-mediated adhesion and the concurrent upregulation of mesenchymal markers such as fibronectin, vimentin, and N-cadherin [135]. Hao et al. showed that Cavin1 overexpression in HCC cells led to increased E-cadherin and reduced N-cadherin, fibronectin, and vimentin expression – strongly suggesting inhibition of EMT [132].

Taken together, these findings indicate that Cavin1 slows EMT progression and may act as a tumor suppressor in HCC by modulating Wnt/β-catenin signaling. Due to its inhibitory effects on tumor growth and metastasis, Cavin1 holds promise as both a prognostic biomarker and a therapeutic target in HCC. However, further research is needed to fully elucidate Cavin1’s additional roles in HCC pathogenesis and to confirm its suitability for clinical application.

#### Cavin1 and glioblastoma (GBM)

GBM is a highly aggressive brain tumor characterized by profound metabolic remodeling, which supports tumor growth and modulates the immune microenvironment [136]. Unlike in non-small cell lung cancer, hepatocellular carcinoma, and colorectal cancer, elevated Cavin1 levels in GBM are associated with a worse prognosis. However, the exact biological role and molecular mechanisms of Cavin1 in GBM metabolism remain incompletely understood.

Studies by Thommen et al. showed that Cavin1 overexpression alters energy metabolism and enhances endocytosis in GBM cells. In in vivo models (GBM xenografts and intracranial tumors), inhibition of cPLA2 reduced tumor development and rescued the Cavin1-induced reduction in CD8⁺ tumor-infiltrating lymphocytes [137]. Later, Yi et al. showed that Cavin1 activates a phospholipid remodeling pathway mediated by cytoplasmic phospholipase A2 (cPLA2), which promotes tumor growth and suppresses anti-tumor immunity. In their studies using primary GBM cell lines, Cavin1 overexpression led to changes in phospholipid composition and increased cPLA2 activity, which was associated with improved cPLA2 protein stability [97, 136].

Interestingly, while Cavin1 overexpression increased cPLA2 levels in N9 and N33 GBM cells, TCGA database analysis did not reveal a correlation at the transcriptional level [136]. qPCR additionally confirmed that cPLA2 mRNA levels remained unchanged, suggesting post-transcriptional regulation. Immunoblotting further indicated that cPLA2 degradation was significantly slower in Cavin1-overexpressing cells, implicating Cavin1 in stabilizing cPLA2 protein [136, 138].

Cavin1-driven metabolic reprogramming in GBM supports tumor survival and immune evasion, encompassing enhanced endocytosis, mitochondrial fatty acid oxidation, and adenosine metabolism [139, 140]. One characteristic metabolic change is the increase in lysophosphatidylcholine (LPC) levels, which modifies membrane fluidity, promotes nutrient import, and boosts endocytosis. These alterations identify Cavin1 and its downstream pathways as promising therapeutic targets, including for adenosine-based immunotherapies [141].

**Fig. 4:**
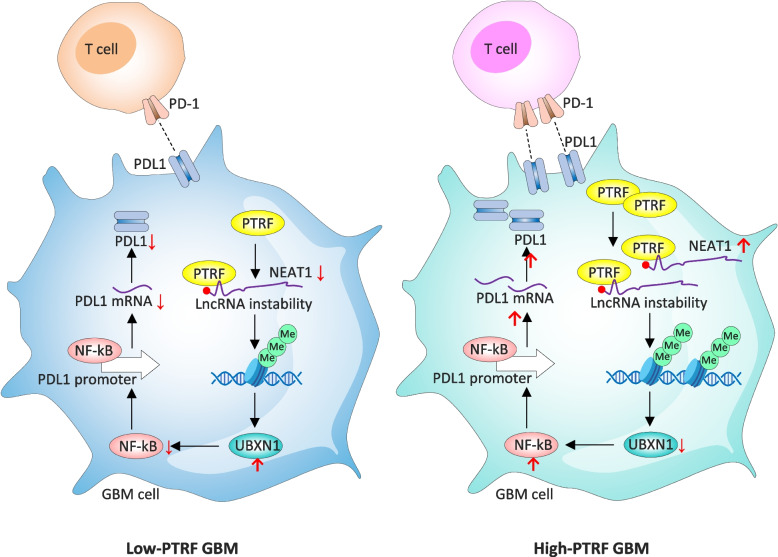
Cavin1 (PTRF) -dependent regulation of immune evasion in glioblastoma via the NEAT1–NF-κB–PDL1 axis, comparing low-PTRF (left) and high-PTRF (right) glioblastoma (GBM) cells and their interaction with T cells through the PD-1/PDL1 immune checkpoint. In the low-PTRF GBM state, reduced Cavin1/PTRF levels lead to decreased stabilization of the long non-coding RNA NEAT1, resulting in increased LncRNA instability. This attenuates NF-κB signaling and lowers transcriptional activation of the PDL1 promoter, leading to reduced PD-1 engagement on T cells, favoring T-cell activation and antitumor immune responses. In contrast, in high-PTRF GBM cells, elevated Cavin1 expression promotes NEAT1 stabilization, facilitating sustained NF-κB activation. Activated NF-κB enhances PDL1 transcription, increasing PDL1 mRNA. High PDL1 levels strengthen PD-1/PDL1 interactions at the T-cell–tumor interface, leading to T-cell inhibition and immune evasion. Additional PTRF-associated regulatory factors (e.g., UBXN1) further modulate NF-κB signaling and chromatin-associated regulatory processes

Beyond metabolism, Cavin1 also influences immune regulation through the stabilization of the long non-coding RNA NEAT1, which in turn upregulates PDL1 (CD274) and NF-κB. Cavin1 enhances PDL1 transcription by activating NF-κB and suppressing UBX Domain Protein 1 (UBXN1) via NEAT1 (Fig. 4). Thommen et al. suggested that this axis facilitates immune evasion and promotes tumor progression [137].

Bioinformatics analysis by Guo et al. revealed a negative correlation between Cavin1 expression and cytotoxic lymphocyte infiltration in GBM, but a positive correlation with fibroblast abundance, highlighting Cavin1’s role in shaping the tumor microenvironment [142]. In GBM, Cavin1 overexpression is typically associated with higher WHO tumor grades and poorer prognosis [42, 143]. Additionally, Cavin1 has been linked to multidrug resistance, co-upregulated with P-glycoprotein in both GBM and breast cancer [97].

Interestingly, the well-known MYC oncogene has also been shown to bind directly to the PDL1 promoter, further contributing to immune evasion in tumor cells. Together, these findings underscore the multifaceted and pro-tumorigenic role of Cavin1 in GBM, including contributions to metabolic adaptation, immune suppression, and therapy resistance.

#### Cavin1 and cutaneous squamous cell carcinoma (cSCC)

Cutaneous squamous cell carcinoma is the second most common form of skin cancer, accounting for nearly 20% of skin tumor-related deaths annually [144–147]. Despite advances in surgery, chemotherapy, and radiotherapy, the prognosis for cSCC remains unsatisfactory, particularly due to rising metastasis and recurrence rate [148, 149].

One possible underlying mechanism of cSCC development involves the regulation of Cavin1 expression by microRNAs (miRNAs) – specifically, miR-217. MiRNAs are short, non-coding RNAs that bind to the 3′ untranslated region (3′ UTR) of target mRNAs, leading to translation inhibition or mRNA degradation [150]. Through regulation of targets like Cavin1, miRNAs influence a range of cellular processes, including migration, invasion, proliferation, differentiation, apoptosis, and tumor development [151–153]. In cSCC, miR-217 is upregulated while Cavin1 is downregulated, supporting an inverse regulatory relationship and suggesting that miR-217 may promote cSCC progression by targeting Cavin1 [150]. Similarly dysregulated (and often extracellular) miRNA profiles are also widely reported in other malignancies, including gastric cancer, hepatocellular carcinoma, breast cancer, and bladder cancer [122, 150, 154, 155]. This strongly suggests that the miR-217/Cavin1 axis might be a promising therapeutic target in cSCC and potentially other aggressive epithelial cancers, offering new opportunities to improve patient outcomes. However, whether these miRNA changes directly converge on Cavin1/PTRF regulation is miRNA- and tumor-type specific and cannot not be assumed without further direct evidences.

#### Cavin1 and rhabdomyosarcoma (RMS)

Rhabdomyosarcoma is a pediatric soft tissue sarcoma characterized by the expression of markers typically found in skeletal muscle, including paired box gene 7 (Pax7), myogenic differentiation protein (MyoD), myogenin, desmin, and muscle-specific actin [107, 156, 157]. In RMS, Cavin1 is expressed in myogenic tumors and primary mouse RMS cultures, as demonstrated by combined in silico and in vitro analyses [131, 156, 157].

In both human and mouse RMS cell cultures, Cavin1 and Caveolin1 have been shown to co-express and form plasma membrane contacts during cell proliferation. Their expression is downregulated during myogenic differentiation. Knockdown experiments revealed that depletion of either Cavin1 or Caveolin1 impairs cell migration and proliferation in human RD and RH30 RMS cell lines. Notably, Cavin1 depletion alone in RD cells led to reduced anchorage-independent growth in soft agar assays [156].

Changes in Cavin1 protein levels – via overexpression or deletion – were found to modulate RMS cell growth, migration, and clonogenic potential [156]. Interestingly, Cavin1 levels in RMS cells remained stable despite gene deletion, indicating the possible presence of compensatory mechanisms probably similar to a “transcriptional adaptation” phenomenon later described by El-Brolosi et al. [158].

These findings suggest that the interaction between Cavin1 and Caveolin1 is a critical driver of cell migration and proliferation in myogenic tumors. Based on this, both proteins are proposed as putative molecular targets for inhibiting proliferation, migration, and anchorage-independent growth in RMS cells [156].

On the signaling level, studies by Jahangiri and Weiss have shown that the PI3K/AKT and MAPK pathways are frequently co-activated in RMS tumors, highlighting their involvement in tumor progression [159]. The possible interplay between Cavin1, Caveolin1, and these pathways in RMS pathogenesis remains to be fully elucidated and represents an important direction for future research.

## CAVIN 2

### Cavin2: gene and protein

Cavin2 is the second major member of the Cavin gene family. It was first identified in 1990 by Burgener et al. due to its high abundance and strong binding affinity for phosphatidylserine in platelets [160, 161]. The protein is encoded by the gene also known as serum deprivation-response protein (SDPR) or phosphatidylserine-binding protein (PS-p68) [161].

**Fig. 5:**
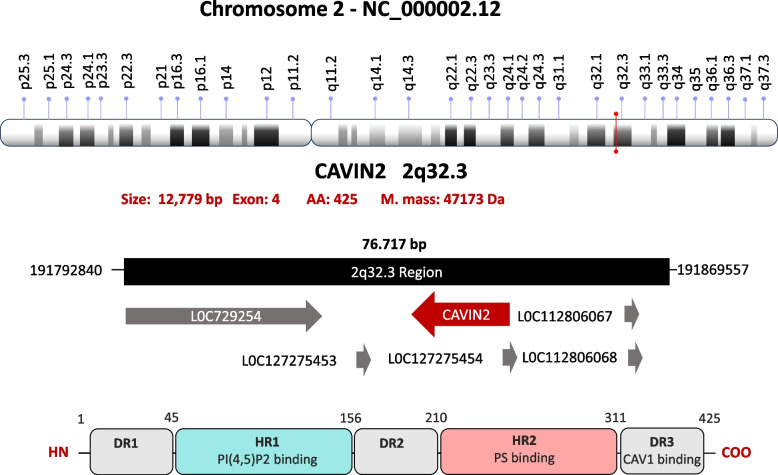
Structure of Cavin2 gene and its transcription product. The upper panel shows the chromosomal localization of CAVIN2 gene (also known as SDPR) on chromosome 2 at band 2q32.3, including its genomic context and orientation within the locus. The CAVIN2 gene consists of four exons and encodes a protein of 425 amino acids with an estimated molecular mass of ~ 47.2 kDa (NCBI Gene ID: 8436). The lower panel illustrates the domain architecture of the Cavin2 protein. Similar to other Cavin family members, Cavin2 is organized into two conserved helical regions (HR1 and HR2) interspersed with three intrinsically disordered regions (DR1–DR3)

Cavin2 consists of 425 amino acids, structurally organized into five major regions (Fig. 5):

- DR1 (45 amino acids)
- HR1 (115 amino acids)
- DR2 (50 amino acids)
- HR2 (110 amino acids)
- DR3 (105 amino acids)

Cavin2 is highly expressed in human endothelial cells and is localized in the cytoplasm, plasma membrane, and caveolae [160]. Its expression is particularly elevated in the heart and lungs, with lower levels detected in the brain, kidney, liver, pancreas, placenta, and skeletal muscle [20]. In developing fibroblasts, Cavin2 is asynchronously upregulated in response to serum deprivation, but not upon contact inhibition. Conversely, Cavin2 expression is downregulated during synchronous re-entry into the cell cycle, indicating a possible role in growth arrest-associated signaling [20].

### Cavin2 function

Similar to Cavin1, Cavin2 plays a central role in caveolar biogenesis and morphological regulation. The loss of Cavin2 leads to a disruption in caveolae formation [36, 162]. Interestingly, the requirement for Cavin2 in caveola formation appears to be tissue-specific: it is essential in lung and fat endothelial cells, but not in heart endothelia, suggesting cell type–dependent roles in caveolar development [36]. Cavin2 also forms direct interactions with Cavin1, facilitating Cavin1 recruitment to caveolar membranes. However, unlike Cavin1, overexpression of Cavin2 can cause caveolae deformation and result in severe tubulation of the plasma membrane [27, 162].

Beyond its structural roles, Cavin2 is involved in signal transduction, particularly in the localization of protein kinase C alpha (PKCα) to caveolae. However, the functional consequences of PKCα overexpression or activation are cell type–dependent and may vary under different physiological or pathological conditions [163]. Additionally, Cavin2 is implicated in regulatory networks such as the nuclear receptor meta-pathway and the glucocorticoid receptor pathway, suggesting broader roles in metabolic homeostasis and endocrine signaling [164, 165].

In the plasma membrane of adipocytes, Cavin2 contributes to the stabilization of the insulin receptor (IR) [20]. This enhances insulin/IR/Akt signaling and promotes adipogenic gene expression, for example in 3T3-L1 adipocytes treated with insulin-containing media [166].

### Cavin2-related disorders

Cavin2 is highly expressed in adipocytes, where mutations in the Cavin2 gene or insufficient Cavin2 expression can impair protein transport, leading to a loss of caveolae formation and the onset of various disorders [36]. The estimated prevalence of the most significant Cavin1-related disorders and the related Cavin1-profiles are shown in Table 3.

**Table 3:** Major diseases associated with Cavin2 and their prevalence

Disease	Worldwide prevalence (recent estimate)	Reference for prevalence	Cavin2 association
Pulmonary injury	~ 64.2 per 100 000	Johnson et al. [167]	Mostly ↓
Heart failure	~ 64 million people worldwide (global estimate)	Savarese et al. [168]	Mostly ↑
Lipodystrophy (generalized forms)	< 1 case per 10 000 000	Purizaca-Rosillo et al. [169]	Mostly ↓

### Cavin2 metabolic/sporadic disorders

#### Cavin2 gene and pulmonary injury

In the lungs, caveolae are particularly abundant in epithelial cells, endothelial cells, adipocytes, and fibroblasts [170, 171], where they mediate critical functions including signal transduction, endocytosis, exocytosis, and molecular transport [172]. Interestingly, the size of Cavin complexes in lung and fat tissues is significantly smaller compared to those in other tissues [170]. In the lung tissues, Cavin1 and Caveolin1, may partially compensate for Cavin2 loss but mostly in the absence of Cavin2, Cavin complexes in the lung become abnormally large and unstable, resulting in shallow caveolae and flattened membrane regions in lung endothelial cells [171]. This phenomenon was also observed in the cultured lung endothelium [41].

A clinically relevant context for Cavin2 function in the lung is lung ischemia—reperfusion injury (LIRI) – a common and severe complication of ischemic lung disease. LIRI is characterized by an imbalance between oxygen supply and metabolic demand, leading to tissue hypoxia, oxidative stress, inflammation, and cell death. The lung’s dual blood supply from the pulmonary and bronchial arteries, and dual oxygen exchange via alveolar and capillary networks, makes the response to ischemia in lung tissue more complex than in other organs.

Although Cavin2 is well-known for its roles in lipid homeostasis, signal transduction, and endocytosis, its specific function in LIRI has only recently begun to emerge. Inflammatory mediators, particularly interleukin-6 (IL-6) and interleukin-10 (IL-10), play a central role in the pathogenesis of LIRI [173]. IL-6 promotes inflammation and cellular injury, while IL-10 exerts anti-inflammatory and cytoprotective effects. In a study by Tang et al., Cavin2 overexpression in rat lung tissue significantly reduced IL-6 and methylenedioxyamphetamine levels, while increasing IL-10 concentrations, thereby attenuating LIRI. These effects were linked to the anti-inflammatory role of Cavin2 and its ability to modulate IL-6 signaling through a feedback regulatory mechanism [171].

Additionally, Cavin2 expression was upregulated under hypoxic conditions, enhancing cellular viability and IL-10 production, and further reducing inflammatory and oxidative markers compared to control conditions. Conversely, Cavin2 knockdown reversed these beneficial effects. Cavin2 overexpression also enhanced the phosphorylation of key signaling molecules including AKT, STAT3, and extracellular signal-regulated kinase ERK1/2, especially under hypoxic conditions, suggesting that Cavin2 modulates survival and inflammatory pathways during pulmonary stress. The IL-6/IL-10 axis remains one of the most studied inflammatory mechanisms related to LIRI [174, 175].

Another related injury is pulmonary arterial hypertension (PAH)—a progressive and often fatal condition characterized by elevated pulmonary arterial pressure and vascular remodeling. Although the molecular basis of PAH is not fully understood, some genes involved in caveolae structure and function – notably Cavin2 and Caveolin1 – have been identified key regulators of caveolae formation and membrane integrity, [176, 177]. In a study by Kasahara et al. [178], simultaneous deficiency of Cavin2 and Caveolin1 led to the complete loss of endothelial caveolae in lung tissue, underlining the cooperative role of these proteins in maintaining caveolar ultrastructure and function.

Given its demonstrated effects on cytokine balance, oxidative stress reduction, and cell survival, Cavin2 emerges as a promising therapeutic target for the treatment of LIRI, PAH and other lung injuries. One proposed approach involves protein delivery systems, such as suitable protein carriers, to modulate Cavin2 activity in the lung [179]. Moreover, computational methods like AdaBoost and the Relevance Vector Machine (AdaRVM) have been employed to identify genetic markers of pulmonary injury [180]. These analyses highlight the Cavin2 gene as a strong candidate involved in pulmonary damage mechanisms and response prediction [176]. However, additional studies are needed to elucidate the precise mechanisms by which Cavin2, along with other Cavins and Caveolins, regulates lung structure, barrier function, and injury responses.

#### Cavin2 and fibroblast role in heart failure

Cavin2 is highly expressed in cardiac fibroblasts, though its precise role in their pathologies such as cardiac fibrosis remains under investigation. Probably, Cavin2 is not essential for caveolae formation in cardiac endothelium [36]. However, a study by Higuchi et al. who used a transverse aortic constriction model, suggested that Cavin2 deficiency inhibits the trans-differentiation of fibroblasts into myofibroblasts by modulating the TGF-β/Smad signaling pathway, a central regulatory axis in fibroblast activation and fibrotic remodeling [181]. This pathway is a current focus of several experimental therapies for heart failure, particularly in conditions involving cardiac fibrosis. The same study demonstrated that fibroblast-specific deletion of Cavin2 led to reduced fibrosis and preserved cardiac function, suggesting that loss of Cavin2 in cardiac fibroblasts may exert protective effects in pressure overload–induced heart failure. The authors further proposed Cavin2 as a potential therapeutic target for the management of cardiac fibrosis [181].

Notably, Cavin1 is in general upregulated in senescent human fibroblasts and aged mouse tissues, indicating a potential compensatory relationship between Cavin family members during cellular aging [180].

#### Cavin2 and lipodystrophy

Individuals with Cavin2 mutations or Cavin2 deficiency, display the disruption of caveolae formation, associated with metabolic abnormalities and lipodystrophic phenotypes. In a study by Yusuke Higuchi et al. [166], Cavin2-deficient mice fed a high-fat diet developed features of metabolic syndrome, including insulin resistance, dyslipidemia, and ectopic fat accumulation. These findings indicated the importance of Cavin2 in maintaining glucose and lipid homeostasis.

**Fig. 6:**
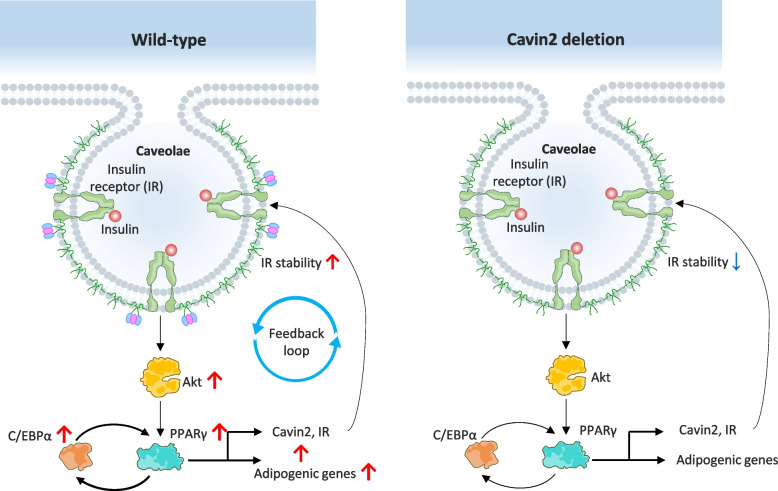
Role of Cavin2 in insulin receptor stability and insulin-dependent adipogenic signaling in wild-type (left) and Cavin2-deficient (right) conditions. In the wild-type state, Cavin2 is highly expressed in adipocytes and localizes to caveolae, where it directly interacts with the β-subunit of the insulin receptor (IR) at the plasma membrane. This interaction stabilizes IR within caveolae, enhancing insulin binding and downstream signaling. Upon insulin stimulation, activated IR promotes Akt (protein kinase B) phosphorylation, which in turn stimulates adipogenic transcriptional agents involving peroxisome proliferator-activated receptor gamma (PPARγ) and basic leucine zipper (bZIP) transcription factor (C/EBPα), leading to increased expression of adipogenic genes. Akt activation also upregulates IR and Cavin2 expression, establishing a positive feedback loop that reinforces insulin sensitivity and adipocyte differentiation. In contrast, in Cavin2 deletion conditions, loss of Cavin2 disrupts IR-caveolae interactions, resulting in reduced IR stability at the plasma membrane. This leads to attenuated insulin-induced Akt signaling, diminished activation of PPARγ and C/EBPα, and impaired expression of adipogenic genes. The absence of Cavin2 also abolishes the reinforcing feedback loop between IR signaling and Cavin2 expression, thereby compromising insulin responsiveness and adipogenic differentiation

Despite high Cavin2 expression in adipocytes, its precise mechanistic role in adipogenesis remains to be elucidated. Studies using knockout (KO) mouse models and 3T3-L1 adipocyte cell lines have shown that Cavin2 directly interacts with the insulin receptor β-subunit (IRβ) at the plasma membrane. This interaction promotes IR stability and enhances insulin/IR/Akt signaling (Fig. 6), promoting the expression of adipogenic genes in 3T3-L1 stimulated with insulin-containing media [166]. Furthermore, activation of IR-mediated Akt signaling enhances the expression of both insulin receptor (IR) and Cavin2, suggesting the existence of a positive feedback loop that reinforces adipocyte insulin sensitivity.

### Cavin2 and cancers

Accumulating evidence highlights Cavin2 as a molecule with tumor-suppressive properties, particularly in the regulation of cell migration, invasion, and proliferation. A significant downregulation of Cavin2 expression has been documented in various malignancies, including breast, kidney, and prostate cancers [182]. Notably, in renal malignancies, reduced Cavin2 serum levels were proposed as a potential diagnostic marker for distinguishing malignant from benign tumors [182].

The chromosome region where Cavin2 gene is located (2q32–33) is frequently associated with loss of heterozygosity and with high recurrence rates in breast cancer [20, 183]. These genetic associations reinforce the relevance of Cavin2 in tumor suppression and genomic stability (Table 4).

**Table 4:** Major cancer types associated with Cavin2 and their prevalence

Disease	Worldwide prevalence (recent estimate)	Reference for prevalence	Cavin2 association
Non-small cell lung cancer (NSCLC)	~ 2.5 million new cases/year	IARC/WHO GLOBOCAN cancer factsheet [123]	Mostly ↓
Hepatocellular carcinoma (HCC)	~ 0.87 million new cases/year	IARC/WHO GLOBOCAN cancer factsheet [123]	Mostly ↓
Oral squamous cell carcinoma (OSCC)	~ 0.39 million new cases/year	IARC/WHO GLOBOCAN cancer factsheet [123]	Mostly ↓

Proper formation and function of caveolae depend on the coordinated interaction between cavins and caveolins. Disruption of the Cavin2/Caveolin1 interaction can lead to caveolar dysfunction and aberrant intracellular signaling, contributing to tumorigenesis. The following sections of this chapter illustrate that reduced Cavin2 expression has also been linked to poor survival outcomes in many cancers, including HCC, glioma, and sarcoma. Ozturk et al. [184] identified Cavin2 as a metastasis suppressor gene in breast cancer, suggesting a broader role in promoting apoptosis of metastatic cells. Similarly, Luo et al. (2019) proposed that Cavin2’s tumor-suppressive functions may extend to multiple malignancies, including gastric cancer [185]. These findings consolidate the potential of Cavin2 as both a prognostic marker and a molecular target in cancer therapy.

#### Cavin2 and non-small lung cancer (NSLC)

Lung cancer remains one of the leading causes of cancer-related mortality globally. Among its histological subtypes, non-small cell lung cancer (NSCLC) accounts for over 85% of cases and includes lung adenocarcinoma (LUAD) and lung squamous cell carcinoma (LUSC) as its principal forms [186, 187]. In both LUAD and LUSC, the expression of caveolins and cavins (especially Cavin2) is significantly downregulated [117, 188, 189].

Functional studies by Peng et al. have demonstrated that Cavin2 overexpression in NSCLC cell lines inhibits migration and invasion, thereby suppressing metastatic potential [190]. In vitro and in vivo models further revealed that ectopic Cavin2 expression leads to G2/M phase cell cycle arrest, reducing proliferation and enhancing chemosensitivity to paclitaxel and 5-fluorouracil – though not to cisplatin [188, 190].

#### Cavin2 and hepatocellular carcinoma (HCC)

**Fig. 7:**
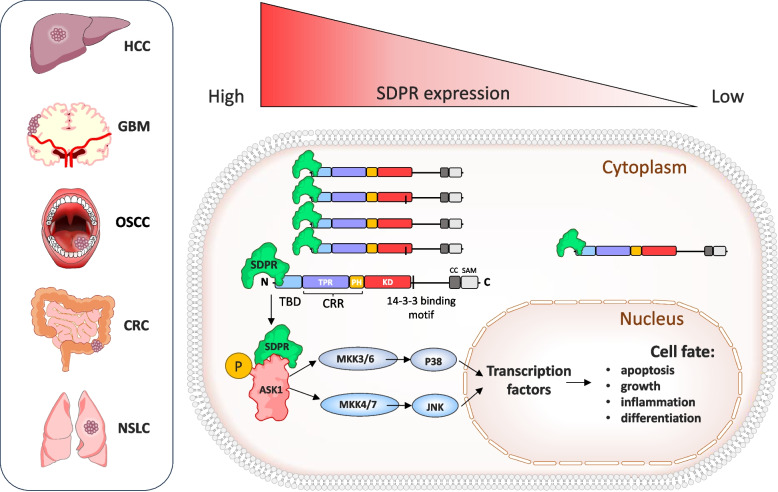
Cavin2 (SDPR)–mediated activation of the ASK1–JNK/p38 apoptotic signaling pathway in tumor cells. Left panel: In cancers such as hepatocellular carcinoma (HCC), glioblastoma (GBM), oral squamous cell carcinoma (OSCC), colorectal cancer (CRC), and non-small cell lung cancer (NSCLC) Cavin2 acts as a tumor-suppressive factor, enhancing apoptotic signaling. Right panel: The context-dependent role of Cavin2 in regulating stress-activated MAPK signaling and apoptosis: In tumor cells expressing high levels of Cavin2, SDPR localizes to the cytoplasm and interacts directly with apoptosis signal-regulating kinase 1 (ASK1). This interaction promotes ASK1 N-terminal dimerization and activation, facilitating downstream phosphorylation cascades involving MAPK kinases (MKK3/6 and MKK4/7). Activated ASK1 subsequently stimulates the stress-activated MAPKs p38 and JNK, which translocate to the nucleus and regulate transcription factors controlling cell fate decisions, including apoptosis, growth inhibition, inflammatory responses, and differentiation. In Cavin2-deficient tumor cells, the absence or strong reduction of SDPR disrupts ASK1 activation and dimerization. As a consequence, downstream JNK and p38 signaling is attenuated, leading to reduced transcriptional activation of pro-apoptotic programs. This impairment favors tumor cell survival, proliferation, and resistance to stress-induced cell death

Early transcriptomic analyses of hepatocellular carcinoma revealed reduced Cavin2 expression in tumor tissues relative to non-tumor counterparts [191, 192]. Although initial mechanistic insights were limited, more recent studies by Chen et al. suggested that Cavin2 exerts anticancer effects by promoting apoptosis via the apoptosis signal-regulating kinase 1 (ASK1)–JNK/P38 signaling cascade [193]. These mechanisms are schematically shown in Fig. 7.

Activation of ASK1 and downstream JNK and p38 MAPK pathways leads to mitochondrial apoptosis and growth inhibition of HCC cells. However, since the ASK1 inhibitor Selonsertib does not completely block Cavin2's pro-apoptotic effects, further research is needed to elucidate potential crosstalk between Cavin2 and other apoptotic pathways [193].

#### Cavin2 and oral squamous cell cancer (OSCC)

**Fig. 8:**
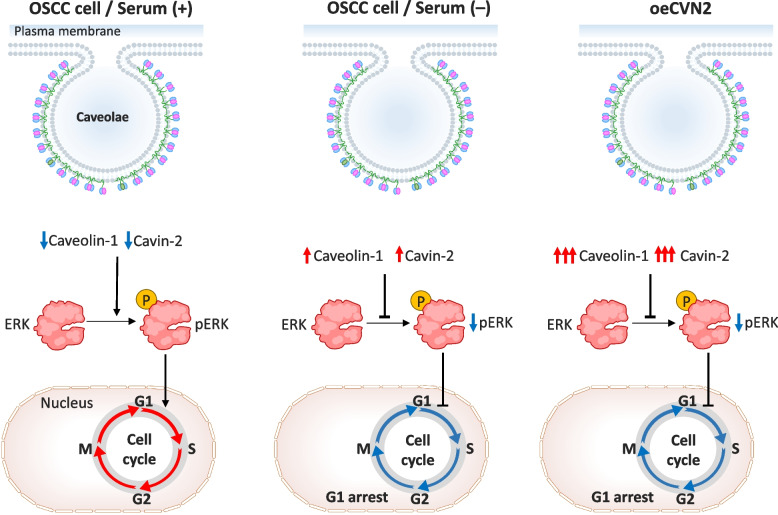
Cavin2–Caveolin1–ERK signaling axis regulating cell-cycle progression in oral squamous cell carcinoma (OSCC). Three panels illustrate schematically how Cavin2 dependent modulation of Caveolin1 expression influences ERK signaling and cell-cycle control in OSCC cells under different conditions. Left panel (OSCC cells, serum +): In the presence of serum, OSCC cells exhibit relatively low levels of Caveolin1 and Cavin2. Under these conditions, caveolae are less stabilized, allowing efficient activation of the ERK pathway, as indicated by increased ERK phosphorylation (pERK). Active ERK signaling promotes progression through the G1/S checkpoint, supporting cell-cycle progression and proliferation. Middle panel (OSCC cells, serum −): Serum deprivation induces upregulation of both Caveolin1 and Cavin2, leading to enhanced caveolae formation and stabilization. Elevated Caveolin1 suppresses ERK phosphorylation, resulting in reduced downstream signaling. This attenuation of ERK activity promotes G1 phase arrest, thereby slowing cell-cycle progression and limiting proliferation. Right panel: Forced overexpression of Cavin2 (oeCVN2) further increases Caveolin1 levels and reinforces caveolae integrity. This strong Cavin2–Caveolin1 interaction markedly inhibits ERK activation, leading to sustained suppression of pERK and robust G1 cell-cycle arrest

Cavin2 contributes to the formation of giant caveolae, which are present in a variety of cell types, including lung, hepatocyte, and oral squamous epithelial cells [36, 162]. In OSCC, both in vitro and in vivo studies have consistently demonstrated that Cavin2 expression is significantly downregulated. In addition, clinically, OSCC patients with low or absent Cavin2 expression tend to present with more aggressive disease phenotypes and worse prognoses [98, 194]. However, its precise molecular mechanisms and clinical relevance in oral squamous cell carcinoma (OSCC) remain insufficiently elucidated [194]. Similar to its behavior in other tumor types, it may be proposed that Cavin2 modulates the expression of Caveolin1, which in turn inhibits extracellular signal-regulated kinase (ERK) signaling – a key pathway implicated in cell proliferation and tumor progression (Fig. 8).

To dissect the regulatory role of Cavin2 in OSCC, quantitative reverse-transcription polymerase chain reaction (qRT-PCR) and immunoblotting were employed by Unozawa et al. in combination with functional studies by using OSCC cell lines engineered to overexpress Cavin2 (oeCavin2). They demonstrated that Cavin2 expression was significantly reduced (p < 0.05) in OSCC tumor tissues compared to adjacent non-malignant tissues [194].

In vitro and in vivo analyses by Changsheng Li et al. and Chettimada S et al. revealed that serum deprivation, a known inducer of Cavin2, led to suppressed OSCC cell proliferation [176, 195]. This effect was attributed to G1 phase cell-cycle arrest, characterized by the downregulation of cyclins and cyclin-dependent kinases (CDKs), and the upregulation of CDK inhibitors such as p21(Cip1) and p27(Kip1) – well-established tumor suppressor proteins.

Notably, phosphorylation of ERK was also markedly reduced, consistent with cell-cycle arrest and proliferation inhibition [194]. Moreover, Cavin2 overexpression enhanced the colocalization and recruitment of Caveolin1 to the plasma membrane, reinforcing caveolar function. This effect has also been observed in cells cultured under serum-deprived conditions, which are known to induce stress responses. Conversely, ectopic overexpression of Cavin2 reduced the proliferative capacity of OSCC cells, predominantly via suppression of the ERK signaling pathway [194].

Together, these findings suggest that Cavin2 may serve as a critical regulator of OSCC progression through the Cavin2/Caveolin1/ERK axis, making it a promising therapeutic target for the development of novel OSCC treatments. Although many studies have reported that serum deprivation upregulates both Caveolin1 and Cavin2 expression [20, 196], more details of their direct interactions in serum-starved cells have yet to be revealed.

## CAVIN3

### Cavin3: gene and protein

Cavin3 is the third identified member of the Cavin protein family, discovered following Cavin1 and Cavin2. It was named for its association with caveolae and is known by several alternative names, such as human SDR-related gene, protein kinase C delta binding protein (PRKCDBP), and serum deprivation response protein (SRBC) [197]. Initially, Cavin3 was identified as a binding partner of protein kinase C delta (PKCδ) and subsequently recognized as an interacting protein of BRCA1 (breast cancer susceptibility gene 1) [198, 199]. The first comprehensive characterization of Cavin3 was carried out by McMahon et al. [200], who localized the protein to caveolae and studied its functional relevance in caveolar biology.

**Fig. 9:**
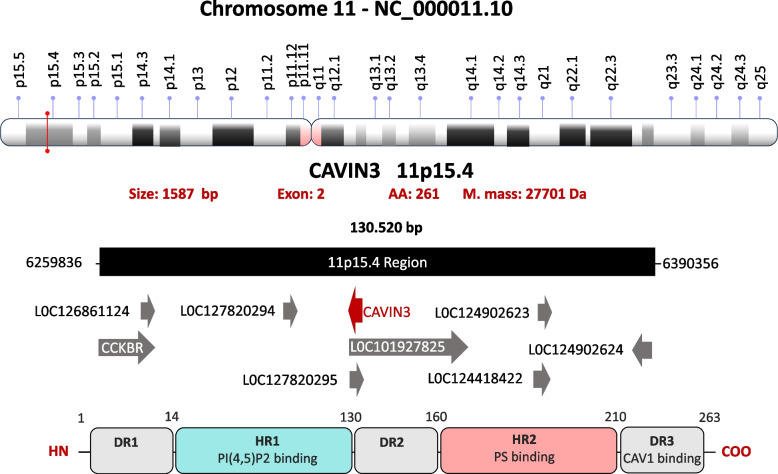
Structure of the Cavin3 gene and its transcription product. The upper panel depicts the chromosomal localization of CAVIN3 gene (also known as SRBC or PRKCDBP) on chromosome 11 at band 11p15.4, including its position and orientation within the genomic locus. The CAVIN3 consists of two exons and encodes a protein of 261 amino acids with an estimated molecular mass of ~ 27.7 kDa (NCBI Gene ID: 284,119). The lower panel illustrates the modular domain organization of the Cavin3 protein. Similar to other members of the Cavin family, Cavin3 contains conserved helical regions (HR1 and HR2) separated by intrinsically disordered regions (DR1–DR3)

The Cavin3 gene is encoded by the SRBC gene, located on chromosome locus 11p15.4 (Fig. 9). The Cavin3 transcript spans 1,587 base pairs, comprising two exons and encoding a 261-amino-acid protein with a molecular mass of approximately 27.7 kDa.

Cavin3 exists in five known isoforms – α, β, γ, δ, and ε – which are collectively referred to as human SRBC (hCavin3) isoforms. These isoforms contain the same five domains (DR1, HR1, DR2, HR2, and DR3) characteristic of the Cavin family [17]. However, the HR2 domain in Cavin3 is less conserved and varies in length, potentially influencing the functional specificity of individual isoforms. In Cavin3, the HR1 and HR2 domains comprise 116 and 50 amino acids, respectively, while the disordered regions DR1, DR2, and DR3 include 14, 30, and 51 amino acids, respectively [17].

Cavin3 is highly expressed in adipose tissue, testes, prostate, colon, mammary epithelium, and cardiac muscle cells. Lower expression levels of Cavin3 are observed in the lung, endometrium, appendix, stomach, spleen, kidney, and heart [201]. Its expression is strongly induced in various cultured cell lines under serum starvation conditions, indicating a role in cellular stress responses. Notably, its expression is independent of Cavin1, distinguishing it from other Cavin family members [37].

At the subcellular level, Cavin3 localizes to the cytoplasm, cytosol, plasma membrane, and caveolae. Interestingly, while Cavin2 is essential for caveolae morphogenesis in endothelial cells of certain tissues, Cavin3 appears to be dispensable for caveolae formation [27]. Nevertheless, its enrichment in human fibroblast caveolae and involvement in protein–protein interactions suggest a more nuanced role in modulating caveolar dynamics and signal transduction, potentially in a tissue-specific or context-dependent manner.

### Cavin3 function

Cavin3 plays diverse roles in cellular signaling and caveolae dynamics. For example, Cavin3 deficiency promotes aerobic glycolysis (Warburg metabolism), accelerates cell proliferation, and inhibits apoptosis. In vivo, the loss of Cavin3 results in cachexia and elevated lactate production [202].

It also presumably modulates the expression and interactions of core circadian clock components, such as period circadian proteins 1 and 2 (PER1/2) and cryptochromes 1 and 2, thereby influencing both the period length and phase of circadian gene expression [170]. Indeed, loss or overexpression of Cavin3 alters the circadian rhythm in fibroblasts by modulating the abundance and interaction of PER and CRY proteins [203].

In general, local cavin composition in the membrane determines caveolar morphology and function. For example, Cavin1 in combination with normal Cavin2 levels produces the classical flask-shaped caveolae, whereas excess Cavin2 results in elongated tubular caveolae. Cavin3, in contrast, is primarily responsible for regulating caveolae budding and endocytic dynamics [35, 43, 162].

Similar to other cavins, Cavin3 is activated under serum starvation, acts as a substrate of protein kinase C and also regulates the Akt and ERK pathways [35, 202]. Cavin3 depletion shifts signaling toward Akt activation (at the expense of ERK), while Cavin3 overexpression shows the opposite effect. In the absence of Cavin3, Akt signaling is activated through PTEN (phosphatase and tensin homolog) suppression and EGR-1 (Early Growth Response Protein 1) inhibition [183, 202].

Mechanistically, Cavin3 promotes ERK signaling by anchoring caveolae to the plasma membrane skeleton through myosin-1c. Disruption of this linkage reduces caveolae number and disconnects the ERK activation module from upstream receptors [202].

Cavin3 contribution to caveolae formation is cell type–specific; for example, smooth muscle endothelial cells seemingly do not require Cavin3, whereas other cell types depend on its presence. Cavin3 also mediates the rapid release and reassociation of caveolae at the cell surface [28, 197].

Under conditions of cellular stress, Cavin3 interacts with BRCA1, stabilizing the protein and facilitating DNA repair [190, 200]). In cells lacking Cavin3, BRCA1 levels are reduced, while Poly ADP-ribose polymerase levels increase as a compensatory mechanism. BRCA1 is phosphorylated by key DNA damage checkpoint kinases – including Ataxia Telangiectasia Mutated (ATM) and Chk1 – allowing cells to repair DNA prior to mitotic entry, thereby enhancing survival following DNA damage [28, 35, 187, 200]. Finally, one of Cavin3 regulatory roles involves the modulation of anti-inflammatory cytokine production via the adenosine A2B receptor pathway [204, 205].

### Cavin3-related disorders

Cavin3 is associated with various pathological conditions, including lipodystrophy, congenital generalized lipodystrophy types 3 and 4 (CGL3/CGL4), and multiple cancers, notably lung and breast cancer (Table 5).

**Table 5:** Major diseases associated with Cavin3 and their prevalence

Disease	Worldwide prevalence (recent estimate)	Reference for prevalence	Cavin3 association
Breast cancer	~ 2.26 million new cases/year	IARC/WHO GLOBOCAN cancer factsheet [123]	Mostly ↓
Lung cancer	~ 2.21 million new cases/year	IARC/WHO GLOBOCAN cancer factsheet [123]	Mostly ↓
Gastric (stomach) cancer	~ 1.09 million new cases/year	IARC/WHO GLOBOCAN cancer factsheet [123]	Mostly ↓
Lipodystrophy (generalized forms)	< 1 case per 10 000 000	Purizaca-Rosillo et al. [169]	Mostly ↓

#### Cavin3 and tumorigenesis

Cavin3 is frequently inactivated in a wide range of human cancers, supporting its role as a tumor suppressor protein. Form the clinical perspective, recent studies by An et al. [206] demonstrated that low Cavin3 expression in breast cancer is correlated with distant metastasis and poor prognosis. Moreover, Cavin3 inactivation has been linked to resistance to oxaliplatin, a chemotherapeutic agent used in colorectal cancer, further highlighting its relevance in tumor progression and treatment response [207].

As mentioned above, in normal tissues, Cavin3 is frequently absent in gastric, lung, and breast cancer cells. This downregulation is often linked to promoter hypermethylation, rather than genetic mutations, pointing to epigenetic silencing as a common mechanism of Cavin3 inactivation. Indeed, promoter hypermethylation of Cavin3 has been detected in 60% of primary breast tumors and 79% of primary lung cancers [199].

In many cells Cavin3 interacts with BRCA1, a key DNA repair protein, and loss of Cavin3 impairs BRCA1-mediated tumor suppression. Although direct mutations in Cavin3 are rare – found only in a few cancer cell lines – its expression is strongly downregulated in various tumors, including those of the lung, stomach, ovary, and colon [208–211]. Loss of heterozygosity (LOH) in the 11p15.5–11p15.4 chromosomal region – where the Cavin3 (SRBC) gene is located near the D11S1323 locus – has been associated with sporadic breast cancer and other tumor types [11].

Further supporting its suppressive role, is the evident link between Cavin3 induction and serum deprivation in cultured cells, strongly suggesting Cavin3 role as a limiting agent, preventing uncontrolled cell proliferation [212].

#### Cavin3 and lipodystrophy

All Cavins together are considered as key regulators of fat metabolism and adipocyte differentiation. Specifically, Cavin3 contributes to adipocyte differentiation by inhibiting TACE-mediated shedding of preadipocyte factor-1 (Pref-1), a key negative regulator that maintains the preadipocyte phenotype [11, 213]. Elevated Cavin3 expression enhances adipogenesis by upregulating adipocyte differentiation markers, whereas reduced Cavin3 impairs this process.

## CAVIN4

### Cavin4: gene and protein

Cavin4 was first identified in muscle cells by Ogata et al., who named it muscle-related coiled-coil protein (MURC) [214]. Cavin4 is primarily localized in the cytoplasm, myofibrils, sarcomeres, membrane, and caveolae. Within the sarcomere, it accumulates at the Z-disc in skeletal muscle and the sarcolemma, but is less abundant in non-muscle caveolae and cytosolic compartments [12, 215].

Although the connection between Cavin4 and caveolae in muscle cells was established later than for other Cavins [9, 21, 214], its localization and associated disease phenotypes strongly resemble those of Caveolin3, particularly in striated muscle cells [216, 217].

**Fig. 10:**
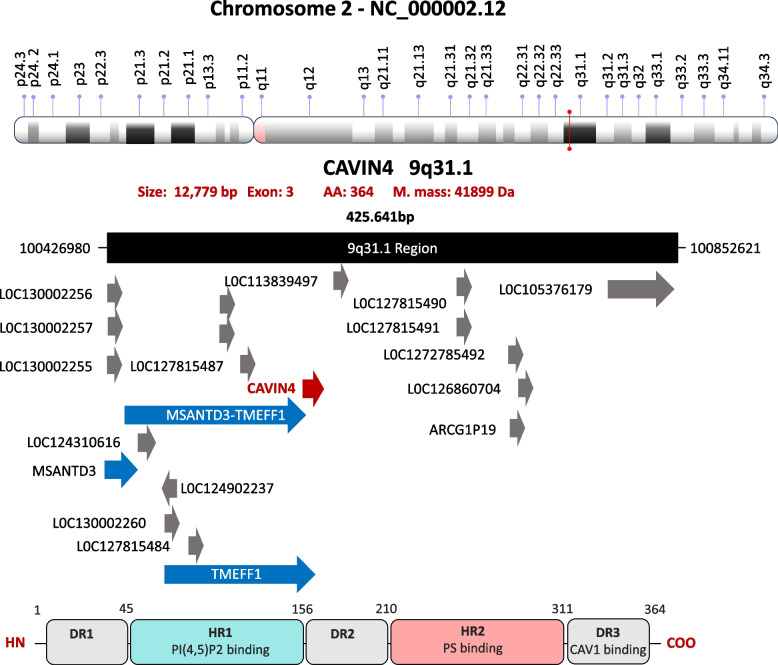
Structure of the Cavin4 gene and its transcription product. The upper panel shows the chromosomal localization of CAVIN4 gene (also referred to as MURC, muscle-restricted coiled-coil protein) on chromosome 9 at band 9q31.1, including its genomic context and transcriptional orientation. According to NCBI annotations (Gene ID: 347,273), the CAVIN4 gene consists of three exons and encodes a protein of 364 amino acids with an estimated molecular mass of ~ 41.9 kDa. The lower panel depicts the domain organization of the Cavin4 protein. Similar to other Cavin family members, Cavin4 contains HR and DR regions. The C-terminal disordered region (DR3) participates in interactions with Caveolin-1 and muscle-specific caveolar components

Cavin4 shares the characteristic five-domain structure of the Cavin protein family [14]: two conserved coiled-coil domains (HR1 and HR2) and three disordered regions (DR1–3). It consists of 364 amino acids, has a molecular weight of approximately 41.9 kDa, and is encoded by a gene spanning 11,523 base pairs (Fig. 10).

### Cavin4 function

Cavin4 is critical for the regulation of caveolae morphology in cardiomyocytes: in cardiac tissue, Cavin4 enhances α1-adrenergic receptor signaling, recruiting ERK1/2 to caveolae and promoting concentric cardiac hypertrophy [218].

Overexpression of Cavin4 causes distension of caveolar structures, whereas its loss impairs cardiac hypertrophy induced by α1-adrenergic receptor stimulation.

Its co-localization with Cavin3 and Caveolin3 in striated muscle cells, along with its exclusive high expression in skeletal muscle, highlights its tissue-specific importance. Cavin4 stabilizes Caveolin3 at the cardiomyocyte membrane, facilitating ERK1/2 signaling and participating in mechanotransduction. In addition, Cavin4 activates the RhoA/ROCK1/2 pathway, promoting transcription of natriuretic peptide A and enhancing myofibrillar organization [215]. Cavin4 can also form complexes with Cavin1–3, reinforcing its collaborative role in caveolae biogenesis and signaling [41, 82]. Cavin4-dependent ROCK and MAPK/ERK pathways regulate hypertrophic responses in cardiomyocytes, culminating in the release of atrial natriuretic peptide, a hallmark of cardiac hypertrophy [37, 214].

### Cavin4 disorders

Cavin4 dysfunction has been implicated in various conditions, including lipodystrophy, congenital generalized lipodystrophy type 4 (CGL4), and congenital myopathies, reflecting overlapping phenotypes across the Cavin family (Table 6).

**Table 6:** Major diseases associated with Cavin4 and their prevalence

Disease	Worldwide prevalence (recent estimate)	Reference for prevalence	Cavin4 association
Heart muscle diseases	overall ~ 0.03–0.2%	Herr et al. [55]	Context-dependent/dual
Immune-mediated rippling muscle disease	Unknown (very rare)	Svahn et al. [219]	Context-dependent/dual

#### Cavin4 and cardiomyopathy

Mutations in the Cavin4 gene have been repeatedly associated with dilated cardiomyopathy [220]. Nakanishi et al. demonstrated that wild-type Cavin4 binds to Caveolin3, anchoring it at the membrane of cardiomyocytes. However, a mutant variant lacking the coiled-coil domain (ΔCC) mislocalizes to the cytoplasm, preventing membrane anchoring of Caveolin3 and reducing its protein levels without altering mRNA [221]. Interestingly, in their studies both wild-type and mutant Cavin4 were able to induce cardiomyocyte hypertrophy, but the ΔCC mutant promoted stronger fetal gene expression, suggesting a more pathological phenotype.

Both Cavin4 deficit and its overexpression can lead to cardiomyopathy, indicating its compex role in physiological regulation and disease progression [218]. Ogata et al. confirmed Cavin4’s interaction with α1-adrenergic receptors s and its ability to enhance ERK activation, while Naito et al. (2015) observed upregulated Cavin4 in response to pressure overload, contributing to pathological remodeling [220]. Although limited, these data suggest that Cavin4 may represent a potential therapeutic target for cardiomyopathies.

#### Cavin4 and rippling muscle disease

Recent findings by Dubey et al. and Svahn et al. suggest that autoantibodies against Cavin4 (Cavin4-IgG) could serve as the first serological biomarker for immune-mediated rippling muscle disease (iRMD). Reduced Cavin4 expression in muscle biopsies from iRMD patients points to its involvement in pathogenesis, especially in paraneoplastic iRMD linked to thymoma, a rare thymic epithelial tumor [46, 219].

These autoantibodies are hypothesized to interfere with Cavin4’s role in caveolae assembly and membrane repair, potentially disrupting muscle homeostasis. While most iRMD cases are acquired and autoimmune, familial forms may arise from genetic mutations affecting Cavin4 or its interaction partners [219].

## Summary

Cavins constitute a small family of caveolae-associated coat proteins that are essential for the formation, stability, and functional specialization of caveolae. Together with caveolins, Cavins1–4 orchestrate caveolar architecture and enable cells to adapt to mechanical, metabolic, and signaling demands in a tissue-specific manner.

The purpose of this review was to highlight the complexity of Cavin roles as dynamic regulators of cellular signaling, metabolism, and stress responses. Cavins1–3 are broadly expressed and participate in diverse physiological processes, including insulin signaling, inflammatory regulation, circadian control, and DNA damage responses, whereas Cavin4 fulfills specialized functions in striated muscle by coordinating caveolar organization, sarcomeric integrity, and mechanosensitive signaling. Importantly, the *functions of Cavins are highly context-dependent*, varying across cell types, tissues, and disease states.

Accumulating evidence links Cavin dysregulation (through genetic mutations, altered expression, epigenetic silencing etc.) to a wide spectrum of human diseases, including lipodystrophy, cardiomyopathies, muscular disorders, and cancer. In oncology, Cavins frequently exhibit tumor-suppressive properties, yet emerging data also reveal *dual or stage-specific roles*, underscoring the complexity of caveolae-associated signaling networks in tumor biology. In metabolic and cardiovascular diseases, Cavins influence insulin sensitivity, fibrosis, inflammatory responses, and tissue resilience to mechanical stress.

From a translational perspective, Cavins are gaining attention as potential biomarkers and therapeutic targets. Their involvement in membrane organization, signaling compartmentalization, and disease-specific pathways positions them as attractive candidates for diagnostic stratification and targeted intervention in caveolae-related disorders. However, significant knowledge gaps remain, particularly regarding Cavin regulation, post-translational modifications, and cell-type-specific functions.

Future studies integrating genetics, epigenetics, advanced imaging, and systems biology approaches will be essential to fully elucidate Cavin-mediated mechanisms and to utilize their therapeutic potential. A deeper understanding of Cavin biology may ultimately enable novel strategies for precision medicine targeting metabolic, cardiovascular, neuromuscular, and oncological diseases.

## Data Availability

No datasets were generated or analysed during the current study.

## Abbreviations

ASK1: Apoptosis Signal-Regulating Kinase 1
ATM: Ataxia Telangiectasia Mutated
AdaRVM: Adaboost and Relevance Vector Machine
Akt: Protein Kinase B
BRCA1: Breast Cancer Gene 1
CGL: Congenital Generalized Lipodystrophy
DR1/2/3: Disordered Region 1/2/3
EGR-1: Early Growth Response Protein 1
ERK: Extracellular Signal-Regulated Kinase
FACs: Focal adhesion complexes
HCC: Hepatocellular Carcinoma
HR1/2: Helical Region 1/2
IL-10: Interleukin 10
IL-6: Interleukin 6
IR: Insulin Receptor
LIRI: Lung Ischemia–Reperfusion Injury
LOH: Loss of Heterozygosity
LUAD: Lung Adenocarcinoma
LUSC: Lung Squamous Cell Carcinoma
MAPK: Mitogen-Activated Protein Kinase
NSCLC: Non-Small Cell Lung Cancer
OSCC: Oral Squamous Cell Carcinoma
PAH: Pulmonary Arterial Hypertension
PKC: Protein Kinase C
PTEN: Phosphatase and Tensin Homolog
Pref-1: Preadipocyte Factor-1
RMS: Rhabdomyosarcoma
ROCK1/2: Rho-Associated Kinases 1 and 2
ROR1: Receptor Tyrosine Kinase-like Orphan Receptor 1
SCLC: Small Cell Lung Cancer
SDPR: Serum Deprivation Response Protein
SRBC: Serum Deprivation Response Protein (human Cavin3)
STAT3: Signal Transducer and Activator of Transcription 3
TACE: TNF-Alpha Converting Enzyme
TNM: Tumor-Node-Metastasis
iRMD: Immune-mediated Rippling Muscle Disease
miRNA: MicroRNA
qRT-PCR: Quantitative Reverse Transcriptase Polymerase Chain Reaction
